# Global genetic diversity of Infectious Salmon Anemia Virus (ISAV) a scoping review protocol

**DOI:** 10.1371/journal.pone.0325115

**Published:** 2025-06-17

**Authors:** Persia Carol Thapa, Ahsan Raquib, Kim Mears, Javier Sanchez, Sonja Saksida, Keith Larry Hammell, Krishna Kumar Thakur

**Affiliations:** 1 Department of Health Management, Atlantic Veterinary College, University of Prince Edward Island, Charlottetown, Canada; 2 Centre for Veterinary Epidemiological Research (CVER), Atlantic Veterinary College, University of Prince Edward Island, Charlottetown, Canada; 3 Robertson Library, University of Prince Edward Island, Charlottetown, Canada; Mansoura University, EGYPT

## Abstract

**Background:**

Infectious salmon anemia virus is one of the most important pathogens responsible for causing infectious salmon anemia in Atlantic salmon (*Salmo salar*). Following its first emergence in 1980s in Norway, it has been reported in several salmon producing countries worldwide, with new variants frequently reported. These variants mostly exhibit differences in segments 5 and 6 of the genome, which contribute to the genetic diversity and variability in virulence. Despite the considerable economic losses associated with ISA, there remains a critical gap in available information on genetic diversity and classification. This study aims to provide a comprehensive and up-to-date synopsis of all known ISAV variants worldwide.

**Methods:**

The Population, Concept, Context approach was used to formulate the research primary question. The primary research question of this review is “What variants of ISAV with respect to segment 5 and 6 has been identified globally in Atlantic salmon?” To address this question, four databases: PubMed, CAB Abstracts via (EBSCO host), Scopus, and the Earth, Atmospheric & Aquatic Science Collection via ProQuest will be used for primary literature search with no language and geographical area restrictions. Studies will be screened using predefined inclusion and exclusion criteria and will be imported in COVIDENCE. Two co-authors will independently screen, extract data, and assess the selected studies. Any discrepancies between the authors will be resolved with the assistance of two other co-authors in each stage of the protocol.

**Discussion:**

To the best of our knowledge, this protocol outlines the first scoping review which will provide insights into the genetic diversity of ISAV, offering a comprehensive overview of the reported variants and their distribution globally. These findings could enhance our understanding of the genetic diversity of the virus, help customize mitigation strategies based on variants involved and provide foundation to develop a universally accepted nomenclature system.

## Introduction

One of the most prominent viral infections in Atlantic salmon (*Salmo salar*) is caused by Infectious Salmon Anemia Virus (ISAV), which has significant negative impact on employment, social and animal welfare, and the global trade [1]. ISAV is an enveloped pleomorphic virus belonging to Orthomyxoviridae family and the only member identified in the genus of Isavirus, represented as a single species currently known as ISAV (*Isavirus salaris*) [2,3]. The Orthomyxoviridae family is characterized by its segmented genome [4]. *Isavirus salaris* contains eight negative sense single stranded RNA segments [5,6]. Sequencing of all eight segments has revealed a total of 10 distinct proteins synthesized by the virus [7,8]. Among the proteins, the surface glycoprotein for fusion (F) and hemagglutinin-esterase (HE) are the most widely studied due to their critical roles in viral pathogenesis [3]. The F protein encoded by segment 5, mediates the fusion of viral and endosomal membranes and the HE protein encoded by segment 6, promotes viral attachment as well as receptor binding and destroying activity [9]. Virulence markers in ISAV have been identified in both segments, arising from insertion or point mutation near the cleavage site of the F gene in segment 5 and deletion in the HE gene in the highly polymorphic region (HPR) of segment 6 [9,10].

The HPR is the most variable region in the entire ISAV genome and is its characteristic feature [3]. The highly polymorphic region when present in full length sequence encodes for 35 amino acids [11,12], and variations in amino acid sequence length in the HPR have been frequently reported [13]. This sequence difference has resulted in the classification of ISAV into distinct groups: European and North American [14,15]. Until now, approximately 30 variants of ISAV have been reported worldwide [1,3,6 2]. These variants are known as HPRΔ or HPR deleted [12] and the variants with long intact ancestral HPR sequence are referred as HPR0 [6,11]. In addition to the deletion in the HPR, mutation from Q266 to L266 or sequence substitution in segment 5 is also present in most of the ISAV-HPR deleted isolates [3,16].

Most importantly, the sequence difference in the HPR has contributed to both the genetic variability of ISAV and differences in the clinical manifestations of the Infectious Salmon Anemia (ISA) outbreaks [7,16]. In 2007, the ISA outbreak in Chile was caused by the ISAV-HPR7b variant [3,16]. This variant was characterized by deletion of 23 amino acids in the HPR region and an insertion of 11 amino acid in segment 5 [16]. The routine surveillance of ISA in 2014 in Faroe Islands discovered low virulent ISAV with 17 amino acids deleted in segment 6 and Q266 to L266 substitution in segment 5 [12]. The first HPR deleted ISAV was detected in salmon flesh rather than viscera in Chinese entry-exit port in 2018, which reported 51 bp deletion in the HPR and no signs of cytopathic effect in cell culture [15]. In an insightful comparative study using 600 sequences of segment 6 from GenBank, a substantial difference in the HPR region of the virulent strain of ISAV was discovered [17].

The classification of ISAV variants is based on deletions in the HPR region [18,19]. For instance, 17, 11, and 23 amino acids are deleted in ISAV-HPR3, HPR-14, and HPR-7b, respectively [16]. In general, variants with the exact same number of deletions in the HPR are grouped together and when a new variant with the same deletion pattern emerges, it is often clustered with the existing variants. This frequently leads to errors and inconsistencies in scientific reporting. These classification inconsistencies present significant challenges when comparing various ISAV isolates, as noted by Kibenge et al. [20]. Cárdenas et al. [21] points out that these inconsistencies complicate epidemiological studies, particularly those conducted by the World Organization for Animal Health (WOAH) that focus on tracing the origins and spread of variants. This situation encourages the development of a universally accepted classification and nomenclature system, beginning with a structured and comprehensive summary of all known ISAV variants worldwide. This could not only improve scientific communication but also progress our understanding of genetics and evolution of ISAV for more effective control and management strategies.

This protocol proposes a first scoping review, to the best of our knowledge, which aims to provide a thorough, and well-structured summary of ISAV variants identified globally. The information summarized from the scoping review allows for comprehensive exploration of the literature where current ISAV variants will be summarized along with additional information, and gaps in understanding the genetic diversity of ISAV will be identified. Moreover, by providing a full spectrum of ISAV diversity we aim to understand the genetic diversity of the virus, which is crucial for risk assessment, evolutionary analysis, evaluate phenotypic variation, and vaccine development [22,23].

### Research question

By applying the Population—Concept—Context (PCC) framework for scoping reviews, primary research question was developed to directly align with and address the objectives of the proposed scoping review [24]. The primary purpose of the review is to answer the following research question: What variants of ISAV have been reported in Atlantic salmon (Population) to date, which amino acids are substituted/deleted/inserted in segments 5 and 6 of these variants (Concept), and what is the global distribution (Context) of these variants?

The secondary purpose of this review is to identify gaps in current understanding that can only be addressed through genomics research, particularly focusing on segments 5 and 6 of the ISAV genome.

## Methods and analysis

This protocol is developed in accordance with the Joanna Briggs Institute (JBI) [24]. The proposed scoping review will be reported based on the PRISMA-ScR (Preferred Reporting Items for Systematic Reviews and Meta-Analyses extension for Scoping Reviews) guidelines [25].

### Eligibility criteria

#### Study designs.

Any observational studies (including cohort studies, case-control studies, and cross-sectional studies) or descriptive studies (case reports, case series) will be included. Review articles, and meta-analysis will be included. Additionally, references from review papers will be evaluated for inclusion in this study.

#### Population.

Studies on either wild or farm raised Atlantic salmon of all life stages will be used. For farm-raised salmon, all types of raising sites: land-based hatcheries for juveniles, saltwater net pens, and land-based grow-out facilities will be included. Experimentally infected Atlantic salmon in laboratory studies (for any purpose) with any type of ISAV variant will also be included. No exclusion criteria will be applied based on the environment where the salmon are raised, the biosecurity method followed by the farm, or differences in management practices.

Fish can be symptomatic or asymptomatic and selected during routine or active surveillance. Similarly, no criteria will be imposed regarding the tissue used for virus isolation or detection. Studies using molecular diagnostic methods such as PCR and sequencing for the detection of ISAV will be included. Any methods used for genomic sequencing (either whole genome sequencing or targeted sequencing) will be included.

#### Concept.

The concept of interest for this scoping review is to explore the mutation pattern/s observed in segments 5 and 6 of ISAV genome. Amino acid (or nucleic acid) sequence of segment 5 and 6 of ISAV will be reported. Studies reporting any other segments except 5 and 6 will not be included. If more than two highly relevant studies reported nucleic acid sequence, then a separate table will be used to distinguish these findings. Additional details such as geographical location, sequencing method, primer used for detection, and associated clinical manifestation will also be included.

#### Context.

There will be no limitation in terms of geographical location of the study. Articles published in any language between 2000 to the 7^th^ of September 2024 will be included.

### Literature search

Four literature search platforms (PubMed, CAB Abstracts with full text via EBSCOhost, Scopus, and the Earth, Atmospheric & Aquatic Science Collection via ProQuest) will be used in this study. Additionally, GenBank, a publicly available and widely recognized genetic sequence database will be used to obtain ISAV sequences. S earches in the literature search platforms will be developed in collaboration with a librarian to identify appropriate keywords, and various subject headings, such as Medical Subject Headings (MeSH) terms (S1 Table). These keywords will be refined for individual databases and Boolean operators “AND” and “OR” will be used for searches. Moreover, the search will include the title and abstract together with keywords or text words together with MeSH terms. A draft PubMed search strategy is presented in S2 Table. After the PubMed search strategy is finalized, the syntax and subject headings will be used for other databases.

### Article type

Primary research published in peer reviewed journals will be included. Studies meeting the following criteria will be excluded: conference proceedings, editorials, letters, and duplicate articles. However, reports, government documents, and grey literature will be searched and added to the final review if relevant information is discovered. As suggested by Donaldson et al. [26], literature search strategy in this study will focus equally on articles and put the same amount of weight on the information provided in each article.

### Screening process

**Fig 1:**
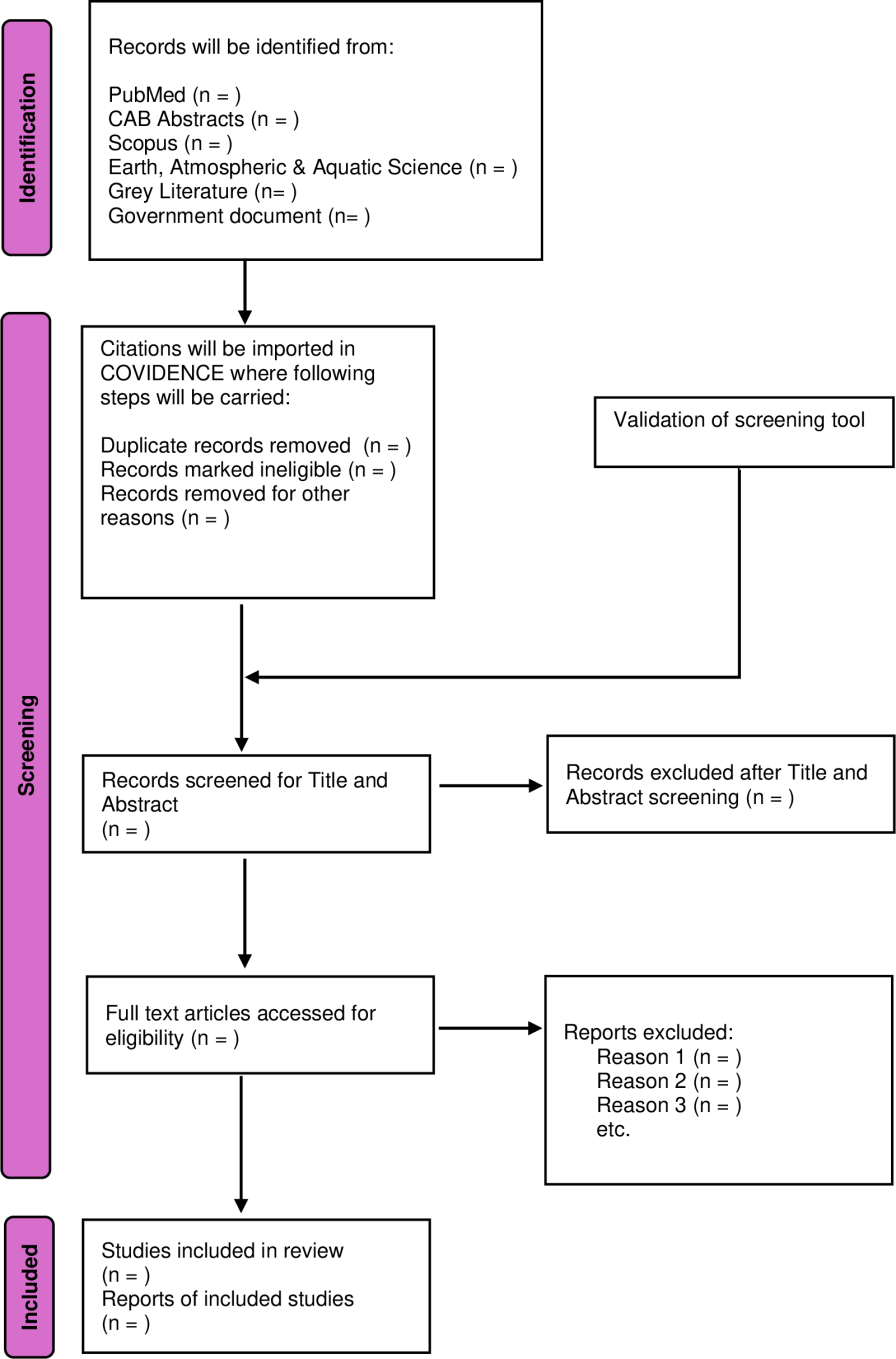
PRISMA 2020 flowchart. The flowchart details the overall process for the scoping review of the global genetic diversity of ISAV. The figure outlines the number of studies identified through databases, screened, excluded, and included at each stage of the review process. At the final stage of full text review, reasons for exclusion will be provided.

The overall screening process is outlined in Fig 1 and will follow the PRISMA guideline [27]. However, before the screening process as suggested by Donaldson & Cooke [26], two co-authors (PCT) and (AR) (referred as the authors henceforth) will randomly subset 10% of articles and review them individually to validate the screening tool as outlined in Table 1. This improves repeatability and consistency in applying the inclusion and exclusion criteria for the articles [26]. The decision to include an article depends on the responses generated, as illustrated in Table 1. An article will be included if all seven screening questions are answered with “Yes” or “Unsure”. It will be excluded if both responses are “No”.

**Table 1:** Screening tool for title and abstract screening.

	Questions	Response (Yes/No/Unsure)
1.	Does the title/abstract describe original research on Atlantic salmon (farmed or wild)?	
2.	Does the title/abstract mention that the study was not a review, systematic review, or meta-analysis?	
3.	Is it clear from the title/abstract that the study was on genetics/genomics of ISAV?	
4.	Does the title/abstract mention that the study focuses on segment 5 and/or 6 of ISAV?	
5.	Does the title/abstract mention that the study focuses on the HPR and/or HPR0 genotype of ISAV?	
6.	Does the title/abstract suggest that the study reports animo acid or nucleotide sequence of segment 5 and/or 6 of ISAV?	
7.	Are ISAV variants or genotypes mentioned in the title/abstract?	

Articles meeting the search criteria will be screened in two stages: first by title and abstract, and then by full text. In the first stage, authors will review titles and abstracts to evaluate the relevance of the selected article with the research question and inclusion criteria. If an article does not have an abstract, then it will be reviewed based on title alone and if relevant, will automatically advance to the next stage of full-text screening. This will be done to ensure that articles that are relevant will not be excluded if the abstract could not be found. In the final stage, authors will analyze the articles in detail, ensuring that only studies meeting all inclusion criteria and relevant evidence are used to derive conclusions for this study.

At each stage, two authors (PCT and AR) will independently review the articles and assess them as “Yes”, “No”, or “Maybe”. If both authors mark an article “Yes”, it will be selected for the proceeding stage. On the contrary, if both authors select “No” for an article, it will be excluded. Article marked as “Maybe” by both authors will advance to the next stage, where a decision will be made after a full text review. Conflicts may arise when an article is marked “Yes” by one author and “No” or “Maybe” by another author. This will be discussed with two other authors (KKT and KLH) to reach a consensus. Furthermore, KKT and KLH will blindly review 10% of randomly selected articles that are excluded from the screening process, as well as articles that pass each stage of review, to ensure consistency and accuracy in the evaluation process [26].

To assess the consistency of the author’s screening decisions, a Kappa statistic will be generated. This indicates the agreement between the two authors [28] and will be reported from Covidence, a tool designed for the step-by-step process of review [29]. In this study we will proceed with reviewing full texts of the selected articles based on screening of titles and abstracts when the Kappa score of greater than 0.8 is achieved, suggesting “almost perfect” agreement between the two authors (PCT and AR) [30].

Additionally, a Kappa score will also be calculated before proceeding to the next stage to ensure consistency between the authors. Following the methodology outlined by Chaudhry & Singh [31], the references used by articles selected for the final stage will be inspected to increase the likelihood of identifying relevant studies that might have been missed during the literature search.

In case full text of eligible studies is not available, the University of Prince Edward Island’s interlibrary loan program will be used. For non-English articles included in the full-text screening, translation of the article will be searched online. If a translated copy cannot be found online, the first author of the publication will be contacted directly to inquire about the availability of a translated version. The first author of the publication will also be contacted if the full text of the article is inaccessible.

### Data management

From each database, articles that pass the search criteria will be downloaded in either PubMed text or RIS text format and uploaded to Covidence. The user-friendly nature of the software allows two or more reviewers to screen relevant articles (Covidence, Veritas Health Innovation, Melbourne, Australia. Available at www.covidence.org). Each screening stage will be recorded, where information such as the total number of records from databases, number of duplicates removed, number of records screened and removed, and final studies in the review will be listed [32].

### Data items and collection strategy

Following information will be extracted from studies selected for full text review. This list may be adjusted as we progress with the review.

- Objective of the study
- Reporting of amino acid insertions in segment 5 along with the sequence (primary outcome)
- Reporting of amino acid deletions in segment 6 along with the sequence (primary outcome)
- Study design
- Geographic location
- Year of identification
- History of ISAV outbreak (location: cage, site, bay, region, country, timeline, year, month, variant reported, sequence of the variant if reported)
- Salmon population (wild or farm)
- Stage of production (if available)
- Sampling strategy (surveillance, research, or outbreak investigation)
- Health status of salmon (symptomatic/asymptomatic/healthy)
- Clinical manifestation (external signs, internal signs, behavioral changes, mortality pattern, or any additional information presented)
- Cage/site level factors (number of fish, water temperature, water quality, stocking density, or any additional information presented)
- Method for both virus isolation and identification
- Diagnostic test used
- PCR primer used
- Sequencing method
- Number of samples tested and number of ISAV positive samples
- Alignment methods
- Phylogenetic analysis information (if provided)
- Name of the variant/s used by authors
- Gaps identified by the author in the article (secondary outcome)

The authors will extract and record all data items in MS Excel, regardless of its original format (either as a figure, table or supplementary information). For amino acid sequences of ISAV, which are frequently presented in tables, the authors will ensure that the sequences are completely extracted, along with the deleted and inserted segments. To ensure consistency and accuracy, KKT and KLH will also randomly and independently check the extracted data. The extracted information will be compared between the two authors, and any inconsistencies will be discussed within the group. All the data generated in this study will be made available in the final review as supporting documents. If the information is unclear and cannot be extracted from a graph or some critical information is missing from the article, the corresponding author will be contacted.

### Data synthesis and presentation

The findings from all the articles that pass the final stage of review will be synthesized and presented in two complementary formats: a) a written narrative and b) graphical representations including figures and charts. The written narrative will incorporate visual elements such as tables and infographics as much as possible. A descriptive summary will also be used to summarize the included studies. This includes the number of articles, year of publication, geographical location, study design, etc. Additionally, this review will also generate a visual heat map to show areas that are well represented (knowledge clusters) and underrepresented (knowledge gaps). This will illustrate regions with high quality topics for research and identify areas that require further studies from a genomics perspective (focusing on segments 5 and 6).

### Amendments to the study protocol

There are no plans to make any changes in the scoping review during the review process. The authors agree to make changes as suggested by the reviewers and revise the manuscript to address these changes. In that case, the authors will clearly document the change and describe the change rationale. The final scoping review will reflect all the changes made.

## Supporting information

S1 Table Keywords and MeSH terms. Identification of appropriate keywords using various subject headings Medical Subject Headings (MeSH) terms individually for Genetic diversity, ISAV, and Atlantic salmon, along with Boolean operators “AND” and “OR”.
(DOCX)

S2 Table PubMed search strategy. A draft of prospective search strategy outlining appropriate keywords, MeSH terms, and Boolean operators that will be used for literature search in PubMed.
(DOCX)

## Abbreviations

F gene/protein: Fusion gene/protein
HE gene/protein: Hemagglutinin-esterase gene/protein
HPR: Highly Polymorphic Region
ISA: Infectious Salmon Anemia
ISAV: Infectious Salmon Anemia Virus
MeSH: Medical Subject Headings
OIE: Office International des Epizooties
PCR: Polymerase Chain Reaction
PRISMA: Preferred Reporting Items for Systematic Reviews and Meta-Analyses
PRISMA-ScR: Preferred Reporting Items for Systematic Reviews and Meta-Analyses extension for Scoping Reviews
WOAH: World Organisation for Animal Health.
