# Supplementary material for: Global genetic diversity of Infectious Salmon Anemia Virus (ISAV) a scoping review protocol
Source: PLoS One. 2025 Jun 17;20(6):e0325115. doi: 10.1371/journal.pone.0325115 (PMC12173177; doi:10.1371/journal.pone.0325115)
Supplement: S1 Table — Identification of appropriate keywords using various subject headings Medical Subject Headings (MeSH) terms individually for Genetic diversity, ISAV, and Atlantic salmon, along with Boolean operators “AND” and “OR”. (DOCX) [file pone.0325115.s001.docx]

**Supplementary file S1: Identification of appropriate keywords using various subject headings (Medical Subject Headings (MeSH) terms.**

| **Genetic diversity** | **ISAV** | **Salmon** |
| --- | --- | --- |
| Gene*[tiab] OR geno*[tiab] OR "Genome"[Mesh] OR "Genetics"[Mesh] OR "Genes"[Mesh] OR "Genetic Markers"[Mesh] OR "Genetic Variation"[Mesh] OR "Sequence variation*"[tiab] OR  "genotype"[MeSH Terms] OR "Viral Proteins"[Mesh] OR "Viral Protein*"[tiab] OR "Segment 6"[tiab] OR  "segment 5"[tiab] OR HPR[tiab] OR HPR0[tiab] OR "Sequence Deletion"[Mesh] OR "Sequence Deletion"[tiab] OR "Amino Acid Sequence"[Mesh] OR "Amino Acid*"[tiab] OR  "Viral Fusion Proteins"[Mesh] OR "Molecular Sequence Data"[Mesh] OR "Molecular Sequence"[tiab] OR "Molecular Structure"[Mesh] OR "Molecular Structure"[tiab] OR "Cell Line"[Mesh] OR  "Cell Line"[tiab] OR "RNA, Viral"[Mesh] OR "Viral RNA"[tiab] OR "RNA Viruses"[Mesh] OR "RNA Virus*"[tiab] OR "Membrane Proteins"[Mesh] OR "Membrane Protein*"[tiab] OR "F protein"[tiab] OR "Mutagenesis, Insertional"[Mesh] OR "Insertional Mutagenesis"[tiab] OR "Mutation"[Mesh] OR "Mutation"[tiab] OR "Evolution, Molecular"[Mesh] OR "Molecular Evolution"[tiab] OR "Recombination, Genetic"[Mesh] OR  "Genetic Recombination"[tiab] OR "hemagglutinin esterase" [Supplementary Concept] OR "hemagglutinin esterase"[tiab] OR "H gene"[tiab] OR "Viral Structural Proteins"[Mesh] OR "Viral Structural Protein*"[tiab] OR "Sequence variation"[tiab] OR "F gene"[tiab] OR "protein gene*"[tiab] OR "Viral Fusion Proteins"[Mesh] OR "Viral Fusion Protein*"[tiab] OR "Hemagglutinins, Viral"[Mesh] OR "Viral Hemagglutinin*"[tiab] OR "Esterases"[Mesh] OR "Esterases"[tiab] | Isavirus*[tiab] OR "Isavirus"[Mesh] OR ISAV[tiab] OR ISA[tiab] OR "infectious salmon anemia virus"[tiab] OR "hemorrhagic kidney syndrome"[tiab] OR "infectious salmon anaemia"[tiab] OR HKS[tiab] | Salmo*[tiab] OR "Salmon"[Mesh] OR "Salmo salar"[Mesh] |
