# Supplementary material for: Global genetic diversity of Infectious Salmon Anemia Virus (ISAV) a scoping review protocol
Source: PLoS One. 2025 Jun 17;20(6):e0325115. doi: 10.1371/journal.pone.0325115 (PMC12173177; doi:10.1371/journal.pone.0325115)
Supplement: S2 Table — A draft of prospective search strategy outlining appropriate keywords, MeSH terms, and Boolean operators that will be used for literature search in PubMed. (DOCX) [file pone.0325115.s002.docx]

**Supplementary file S2: A draft of prospective search strategy for PubMed.**

((Gene*[tiab] OR geno*[tiab] OR "Genome"[Mesh] OR "Genetics"[Mesh] OR "Genes"[Mesh] OR "Genetic Markers"[Mesh] OR "Genetic Variation"[Mesh] OR "Sequence variation*"[tiab] OR "genotype"[MeSH Terms] OR "Viral Proteins"[Mesh] OR "Viral Protein*"[tiab] OR "Segment 6"[tiab] OR "segment 5"[tiab] OR HPR[tiab] OR HPR0[tiab] OR "Sequence Deletion"[Mesh] OR "Sequence Deletion"[tiab] OR "Amino Acid Sequence"[Mesh] OR "Amino Acid*"[tiab] OR "Viral Fusion Proteins"[Mesh] OR "Molecular Sequence Data"[Mesh] OR "Molecular Sequence"[tiab] OR "Molecular Structure"[Mesh] OR "Molecular Structure"[tiab] OR "Cell Line"[Mesh] OR "Cell Line"[tiab] OR "RNA, Viral"[Mesh] OR "Viral RNA"[tiab] OR "RNA Viruses"[Mesh] OR "RNA Virus*"[tiab] OR "Membrane Proteins"[Mesh] OR "Membrane Protein*"[tiab] OR "F protein"[tiab] OR "Mutagenesis, Insertional"[Mesh] OR "Insertional Mutagenesis"[tiab] OR "Mutation"[Mesh] OR "Mutation"[tiab] OR "Evolution, Molecular"[Mesh] OR "Molecular Evolution"[tiab] OR "Recombination, Genetic"[Mesh] OR "Genetic Recombination"[tiab] OR "hemagglutinin esterase" [Supplementary Concept] OR "hemagglutinin esterase"[tiab] OR "H gene"[tiab] OR "Viral Structural Proteins"[Mesh] OR "Viral Structural Protein*"[tiab] OR "Sequence variation"[tiab] OR "F gene"[tiab] OR "protein gene*"[tiab] OR "Viral Fusion Proteins"[Mesh] OR "Viral Fusion Protein*"[tiab] OR "Hemagglutinins, Viral"[Mesh] OR "Viral Hemagglutinin*"[tiab] OR "Esterases"[Mesh] OR "Esterases"[tiab]) AND (Isavirus*[tiab] OR "Isavirus"[Mesh] OR ISAV[tiab] OR ISA[tiab] OR "infectious salmon anemia virus"[tiab] OR "hemorrhagic kidney syndrome"[tiab] OR "infectious salmon anaemia"[tiab] OR HKS[tiab])) AND (Salmo*[tiab] OR "Salmon"[Mesh] OR "Salmo salar"[Mesh])
